# Genetic and Methylome Variation in Turkish *Brachypodium Distachyon* Accessions Differentiate Two Geographically Distinct Subpopulations

**DOI:** 10.3390/ijms21186700

**Published:** 2020-09-13

**Authors:** Aleksandra Skalska, Christoph Stritt, Michele Wyler, Hefin W. Williams, Martin Vickers, Jiwan Han, Metin Tuna, Gulsemin Savas Tuna, Karolina Susek, Martin Swain, Rafał K. Wóycicki, Saurabh Chaudhary, Fiona Corke, John H. Doonan, Anne C. Roulin, Robert Hasterok, Luis A. J. Mur

**Affiliations:** 1 Plant Cytogenetics and Molecular Biology Group, Institute of Biology, Biotechnology and Environmental Protection, Faculty of Natural Sciences, University of Silesia in Katowice, 40–032 Katowice, Poland; askalska@us.edu.pl; 2 Department of Plant and Microbial Biology, University of Zürich, 8008 Zürich, Switzerland; christoph.stritt@uzh.ch (C.S.); michele.wyler@botinst.uzh.ch (M.W.); anne.roulin@botinst.uzh.ch (A.C.R.); 3 Institute of Biological, Environmental and Rural Sciences (IBERS), Aberystwyth University, Aberystwyth SY23 3DA, UK; hew05@aber.ac.uk (H.W.W.); mts11@aber.ac.uk (M.S.); 4 The John Innes Centre, Norwich Research Park, Norwich NR4 7UH, UK; martinj.vickers@gmail.com; 5 Shanxi Agricultural University, Taigu, Shanxi 030801, China; hanjiwan@sxau.edu.cn; 6 Department of Field Crops, Faculty of Agriculture, Tekirdag Namik Kemal University, Suleymanpasa 59030, Tekirdag, Turkey; mtuna@nku.edu.tr; 7 Tekirdag Anatolian High School, 59030 Suleymanpasa, Tekirdag, Turkey; glsvs@yahoo.com; 8 Department of Genomics, Institute of Plant Genetics, Polish Academy of Sciences, 60–479 Poznan, Poland; ksus@igr.poznan.pl; 9 Applied Omics—Rafał Wóycicki, 31–510 Kraków, Poland; rafal.woycicki@appliedomics.com; 10 School of Biosciences, Cardiff University, Cardiff CF10 3AX, UK; s.chaudhary84@outlook.com; 11 National Plant Phenomics Centre, Institute of Biological, Environmental and Rural Sciences (IBERS), Aberystwyth University, Aberystwyth SY23 3EB, UK; fic5@aber.ac.uk (F.C.); john.doonan@aber.ac.uk (J.H.D.)

**Keywords:** Brachypodium, DNA methylation, drought, flowering, genome, phenomics

## Abstract

*Brachypodium distachyon* (Brachypodium) is a non-domesticated model grass species that can be used to test if variation in genetic sequence or methylation are linked to environmental differences. To assess this, we collected seeds from 12 sites within five climatically distinct regions of Turkey. Seeds from each region were grown under standardized growth conditions in the UK to preserve methylated sequence variation. At six weeks following germination, leaves were sampled and assessed for genomic and DNA methylation variation. In a follow-up experiment, phenomic approaches were used to describe plant growth and drought responses. Genome sequencing and population structure analysis suggested three ancestral clusters across the Mediterranean, two of which were geographically separated in Turkey into coastal and central subpopulations. Phenotypic analyses showed that the coastal subpopulation tended to exhibit relatively delayed flowering and the central, increased drought tolerance as indicated by reduced yellowing. Genome-wide methylation analyses in GpC, CHG and CHH contexts also showed variation which aligned with the separation into coastal and central subpopulations. The climate niche modelling of both subpopulations showed a significant influence from the “Precipitation in the Driest Quarter” on the central subpopulation and “Temperature of the Coldest Month” on the coastal subpopulation. Our work demonstrates genetic diversity and variation in DNA methylation in Turkish accessions of Brachypodium that may be associated with climate variables and the molecular basis of which will feature in ongoing analyses.

## 1. Introduction

*Brachypodium distachyon* (hereafter Brachypodium) is a well-established grass model species, to be found mostly in countries bordering the Mediterranean. With its relatively small (~270 Mb) [1] nuclear genome it possesses most of the genomic and functional genomic infrastructure seen in *Arabidopsis thaliana* (hereafter Arabidopsis) [2,3]. The model has been developed to foster our understanding of such as grass cell wall biology e.g., [4] and flowering control e.g., [5]. Brachypodium has also been the subject of many studies into drought e.g., [6], salt and cold e.g., [7] and defining tolerance mechanisms of relevance to grasses and cereal crops.

Although ecologically important, until recently, the geographical variation of Brachypodium has been relatively poorly characterized [2]. Genome-wide variation in single nucleotide polymorphisms (SNPs) revealed a link to the geographical locations of accessions, with SNP variation suggesting a total of 15 genes being significantly related to environmental adaptation [8]. Genotyping by sequencing of >1400 accessions allowed the definition of a geographical split between the Western and Eastern Mediterranean populations but within each population there existed the same A and B subspecies. It was proposed that both subspecies re-colonized the Mediterranean basin after glaciation followed by lesser, allopatric genetic diversification [9]. Further information on variation was revealed from the pan-genome based on the resequencing of 54 inbred Brachypodium accessions. This study focused on existing inbred Turkish (T^+^) and Spanish (S^+^) accessions and identified 3,933,264 high-confidence SNPs. Phenotypically, the populations could be split between Extremely Delayed Flowering (EDF^+^) phenotype, which was most common in the T^+^ populations, and those where flowering was more rapid [10]. This genomic and phenotypic variation in the Turkish population was not associated with any precise geographical areas within Turkey.

Recently, increasing attention has focused on the possible contribution of DNA methylation, as a component of the epigenomic variation in response to environmental adaptation. DNA methylation forms distinct patterns on cytosines; 5′C-phosphate-G3′ (CpG), CHG, and CHH contexts (where H is any nucleotide except for G) [11] which together represent the methylome. Revealing such variations could identify features linked to the evolution of ecotypes [12]. This idea is supported by reports of stress-associated changes in the epigenome. For example, analyses of methylation sensitive amplified fragment length polymorphisms have suggested that whole methylome variation in plants correlates with environmental variables such as salt concentration [13] or the degree of plant isolation [14,15]. Now, with the widespread use of bisulfite sequencing (BS-Seq), finer scale mapping of the methylome is possible and responses to stress have been further suggested in, for example, transgenerational acquired resistance to disease [16].

Arabidopsis has proven to be especially useful in examining epigenetic variation related to the environment. A study of 150 Swedish Arabidopsis accessions demonstrated considerable epigenomic variation, particularly around transposable elements (TEs), when Arabidopsis was grown at 10 °C or 16 °C [17]. When the geographical origins of the accessions were considered, variation correlated with the relative degree of photosynthetically active radiation in spring and the strongest association was between CpG methylation and latitude [17]. The 1001 epigenome project assessed a global collection of Arabidopsis and further indicated that variation in methylation was related to geographical origin [18]. The methylome appeared to be shaped greatly by the genomic architecture of TEs which can influence the expression of nearby genes. Examining variably expressed genes indicated the prominence of genes linked to defense; for example, resistance genes, which were enriched in methylation in CpG and CHG and/or CHH contexts [18]. In segregating populations derived from an Arabidopsis Cvi × *Ler* cross, phenotypic differences, e.g., flowering time, were linked to patterns of DNA methylation [19]. Other analyses have linked differentially methylated regions with patterns of glucosinolate production [20]. In Brachypodium, the methylation patterns of seven inbred resequenced lines were found to correlate with the degree of genetic variation [21]. A recent more extensive assessment of DNA methylation was based on 83 inbred Turkish accessions and some invasive Australian accessions [22]. Considerable phenotypic variation was mostly correlated with SNP and DNA methylation patterns. There were some limited effects of CG methylation on certain phenotypic features [23].

In this current study, we adopted a different strategy to assess the variation in genomic sequences, DNA methylation and phenotypes in Brachypodium. Thus, we established a new bespoke collection of 55 Brachypodium accessions from Turkey, a center of Brachypodium diversification [3]. The sampling sites were selected to conform to the distinctive climatic regions of Turkey. Crucially, in order to maintain variation in DNA methylation, the seeds were used immediately in experimentation without inbreeding. This makes our Brachypodium collection unique. Seeds were transferred to the UK, and germinated under controlled environment conditions to avoid the introduction of variation in DNA methylation due to intergenerational changes [24]. We observed two major subpopulations in Turkey which could be distinguished based on variation in genome sequences and DNA methylation. Further, our study suggested that these subpopulations can be geographically separated into those from “coastal” and “central” regions. Physiologically, these subpopulations were distinguishable based on flowering requirements and relative drought tolerance as defined using phenomic approaches. This represents a foundational study based on which the nature of possible adaptive changes will be defined. 

## 2. Results

### 2.1. Genomic Diversity Reveals Two Subpopulations in Turkey

To establish if genomic variation can be linked to climatic variables, a new collection of Brachypodium accessions was required where seed sampling reflected the different prevailing conditions. Köppen climate classifications can be used to divide Turkey into seven different climatic environments [25]. These were used to define our sampling strategy where 12 accessions were obtained from the regions designated 1a, 1c, 2, 3 and 4 (Supplementary Materials Figure S1). On advice from local collectors, region 1d was amalgamated with 3, as the collecting sites on the 3/1d border could not be precisely geographically defined. The collection sites are listed in Table 1. No Brachypodium accessions could be collected from region 1b as this was a high-altitude region where Brachypodium is not commonly found [26]. 

**Figure 1:**
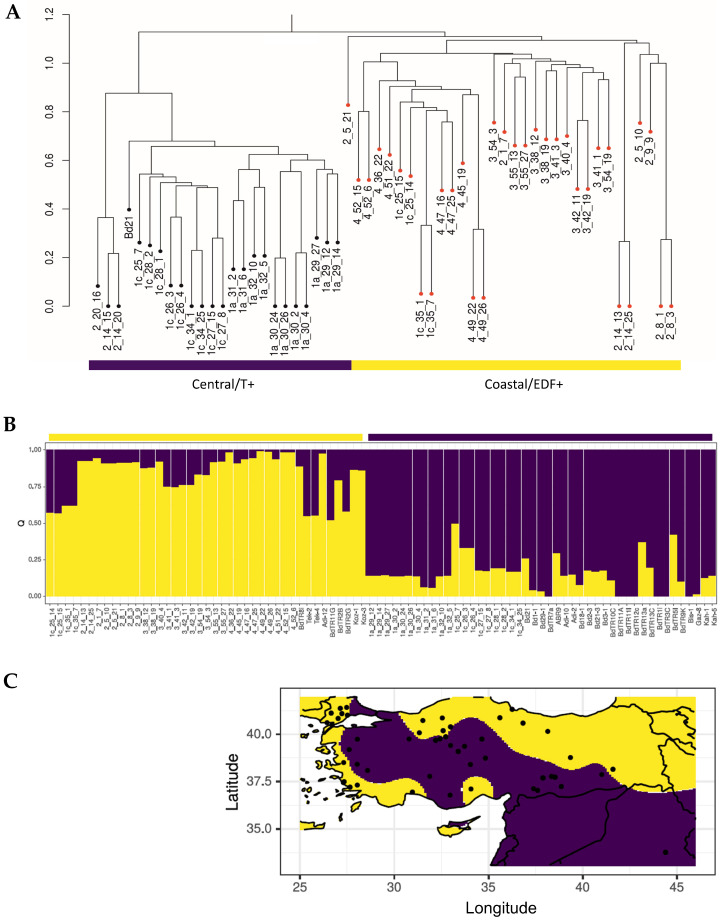
(**A**) Genetic diversity of Brachypodium germplasm from different environmental regions of Turkey as indicated using hierarchical clustering. (**B**) Ancestry coefficients were estimated with TESS3 two ancestral groups amongst Brachypodium accessions (listed in Supplementary Materials Table S1) and (**C**) mapped to Turkey by purple and yellow colors. The yellow and purple horizontal regions indicate accessions corresponding to the ancestral groups and geographically distinguishable as coastal and central subpopulations, respectively. The correspondence between the coastal and central subpopulations and the previously defined Extremely Delayed Flowering (EDF^+^) and Turkish (T^+^) populations [10], respectively, is indicated.

Following genomic sequencing of each accession and Bd21 as the canonical reference, the newly obtained data were joined with publicly available sequencing data for accessions from Spain and Turkey and individual accessions from France (ABR2) and Slovenia (ABR9) [10]. From the initial 8,556,181 hard-filtered SNPs identified among the 111 accessions, a set of 5,792 unlinked SNPs at synonymous positions were obtained for the characterization of genetic structure. Initial analyses compared the genetic variation in the Turkish accessions using a cluster analysis in SNPRelate package implemented in R, this indicated two main groups (Figure 1A). One branch contained all of the accessions from regions 3 and 4, but also some from regions 1c (1c_25_14, 1c_25_15, 1c_35_1, 1c_35.7). Conversely, the other group contained all the accessions from region 1a, some from 1c and included Bd21, which originated from Iraq and was therefore geographically close to the 1a/1c regions. Reflecting their geographical origins, we designated these groups as coastal (regions 2, 3 and 4) and central (regions 1a and 1c) subpopulations. Within the coastal subpopulation, we observed a separate clade of accessions 2_20_16, 2_14_15 and 2_15_20. Principal component analyses (PCA) with these SNPs shows that the two subpopulations of the newly collected accessions aligned with the T^+^ and the EDF^+^ previously defined by Gordon et al. [10] (Supplementary Materials Figure S2). Those accessions from the 1c region which were found within the coastal subpopulation (1c_25_14, 1c_25_15, 1c_35_1, 1c_35_7) were genotypically EDF^+^. Surprisingly, accessions 2_20_16, 2_14_15 and 2_15_20 belonged to the S^+^ cluster, which otherwise comprises only accessions from Spain and France (Supplementary Materials Figure S2). These various designations based on genetic, phenotypic and geographical variation are listed in Supplementary Materials Table S1.

In order to obtain a more detailed picture of the geographic distribution of the two genetic clusters present in Turkey, TESS3 was used to estimate ancestry components and project them onto a map (Figure 1B). Model fit improves as the number of ancestral populations (K) in the model increases, showing that population structure is strongly hierarchical (Supplementary Materials Figure S3). After K = 2, however, increase in model fit is marginal, indicating that a K of 2 describes the most important level of population subdivision. In agreement with the results obtained using PCA (Supplementary Materials Figure S2), at K = 2 one subpopulation corresponds to the EDF^+^ cluster and contains all the accessions from the Köppen regions 3 and 4, but it should be noted some from regions 1c and 2 (Figure 1C, yellow). Conversely, the other subpopulation corresponds to the T^+^ cluster and contains all accessions from region 1a, some from 1c and also Bd21 (Figure 1C, purple). Previous studies did not observe this geographic pattern because the coastal/ EDF^+^ subpopulation was hugely under-represented: with only seven accessions of this subpopulation being sequenced before, compared to 27 of the central/ T^+^ subpopulation [10].

### 2.2. Whole Genome Methylation Assessments Also Indicate Two Subpopulations in the Turkish Population of Brachypodium

**Figure 2:**
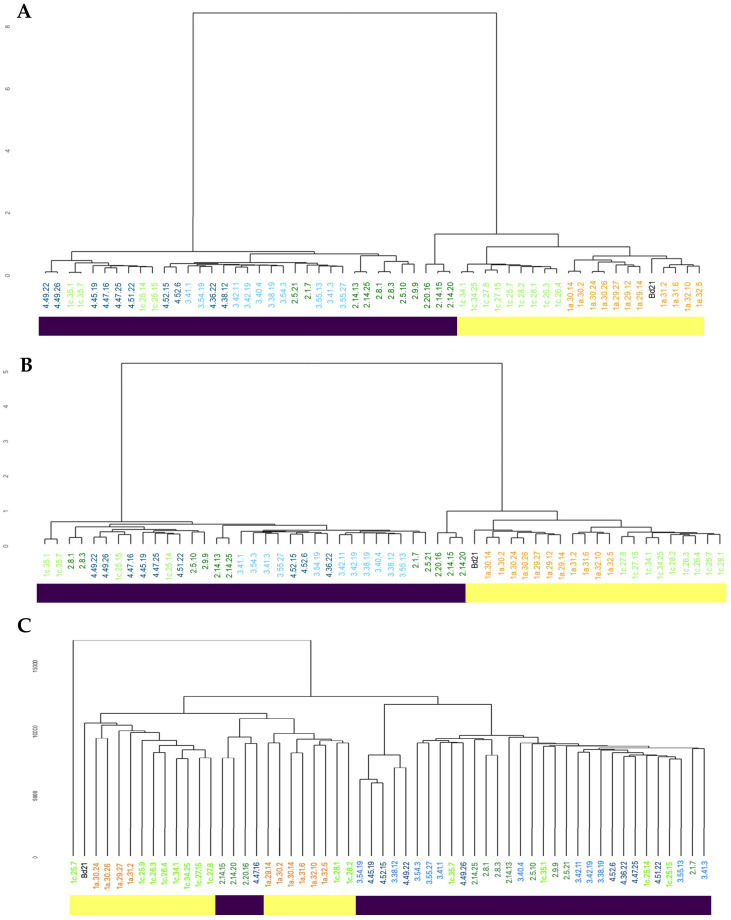
Hierarchical clustering analysis showing variation in whole genome (**A**) CpG, (**B**) CHG and (**C**) CHH methylation patterns based on the similarity of the accession’s methylation profiles. Accessions from particular regions are color-coded (1a—orange, 1c—light green, 2—dark green, 3—light blue, 4—dark blue). Bd21 (from Iraq) is indicated in black. The yellow and purple horizontal bars indicate accessions broadly classified as central and coastal subpopulations, respectively. Region 1c accessions (1c_25_14, 1c_25_15, 1c_35_1, 1c_35_7) with an Extremely Delayed Flowering (EDF^+^) [10] genotype are also located with the coastal subpopulation clade and are given that classification. Although classified as part of the coastal subpopulation the S^+^ genotype accessions 2_14_15, 2_14_20, 2_20_16, were found in distinct clades in each methylation context; especially in CHH (**C**).

We next assessed how the methylome could also vary across the Turkish regions that were sampled. DNA extracted from sample T_0_ plant material was subjected to BS-Seq to reveal genome-wide cytosine methylation. The extent of methylation in different contexts across the population is given in Supplementary Materials Table S2. This indicated that CpG was the most common form of methylation (ranging between 54.3 and 67.8% of bases), followed by CHG (ranging between 28.4 and 43.7% of bases) and CHH (ranging between 1.3 and 10.2% of bases). Visualization of the variation in CpG methylation at a genomic level by hierarchical cluster analysis indicated geographic region-specific clustering in the Turkish accessions (Figure 2A). These clusters exhibited a significant geographic bias with the 1a southern group including the reference accession Bd21 from Iraq. Crucially, the major separation in CpG was between regions which represented the coastal and central subpopulations. With the other methylation contexts; CHG and CHH, also suggest epigenomic separation of the coastal and central subpopulations. This was most prominent in the CHG context compared to CHH (Figure 2B,C). Those EDF^+^ genotypes from region 1c (1c_25_14, 1c_25_15, 1c_35_1, 1c_35_7) also exhibited methylomic variation which placed them in the same coastal subpopulations. Further, in all contexts, the S^+^ accessions 2_20_16, 2_14_15 and 2_15_20 in our collection had distinctive features of their methylome compared to the other Turkish accessions. Therefore, genetic and methylomic variation in the Turkish accessions were closely aligned.

### 2.3. Phenomic Assessment of the Turkish Brachypodium Collection

**Figure 3:**
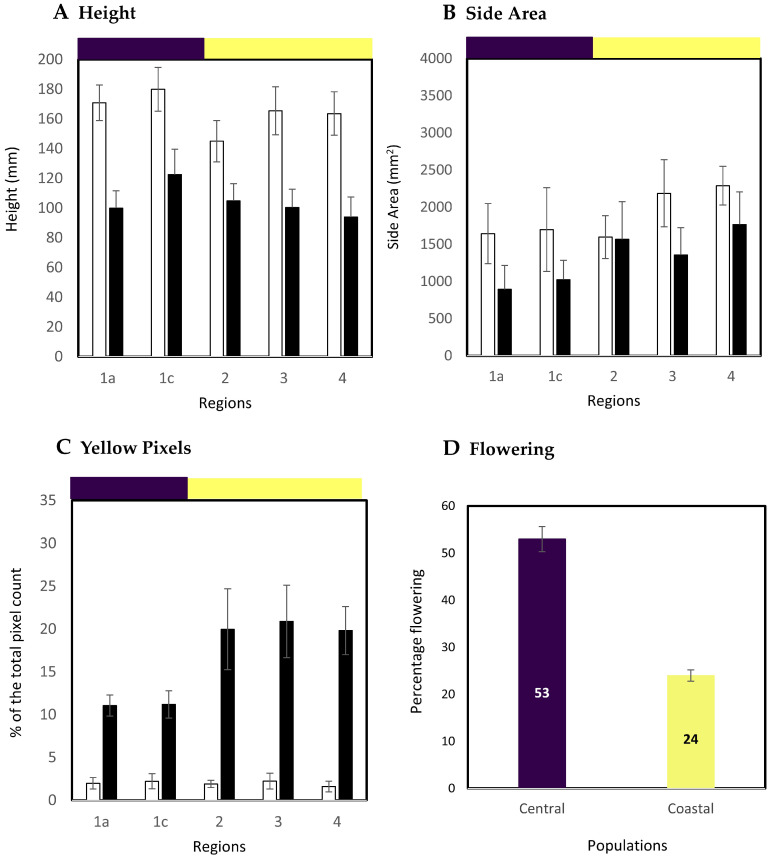
Phenotypic variation in the Turkish collection of Brachypodium. Brachypodium accessions (*n =* 8 plants) were vernalized for six weeks at 4 °C before being transferred to 22 °C and either maintained with full watering (*n =* 4 plants, white bars) or at 15% soil water content (*n =* 4 plants, black bars). At 12 d the plants were imaged at the National Plant Phenomics Centre, Aberystwyth, UK, where (**A**) height and (**B**) side area were derived. Data are grouped based on regional origins (1a, 1c, 2, 3, 4); (**C**) yellow pixels were extracted from the images of plants. Pixel data are presented as % of the total pixel count for the whole plant. The purple and yellow horizontal bars on (**A**–**C**) indicate data from accessions sampled from central (1a, 1c) and coastal (2, 3, 4) subpopulations, respectively; (**D**) after a further eight weeks the percentage of control plants originating from the coastal and central subpopulations which had flowered was measured.

Computerized image analysis approaches were employed to assess phenotypic variation in the sampling sites. Seeds (*n =* 8) from each accession were sown and at two weeks after sowing were vernalized at 4 °C for six weeks. After two weeks of growth at 22 °C (*n =* 4) plants of each accession were exposed to drought targeting 15% soil water content (SWC) over a period of 12 days. We had previously shown that this level of SWC was sufficient to impose drought stress on a diversity collection of Brachypodium accessions [6]. The remaining (*n =* 4) control plants were watered as normal. RGB images were obtained for plants and assessed for height and area as estimated from side view images (Supplementary Materials Figure S4) to provide a proxy for growth [27]. Phenotypic data (Supplementary Materials Table S3) obtained for individual accessions are provided in Supplementary Materials Figure S5). When accessions were considered based on regions, both plant height and side area were significantly reduced (*p <* 0.05) by drought treatment (Figure 3A,B). However, although there was considerable variation in accession height and area, no significant differences (*p* = 0.92) were observed between the different regions or the previously defined population groups.

Pixel colors were extracted from the images and the percentage of yellow pixels (indicative of stress associated leaf senescence) was significantly less in plants from region 1a and 1c as compared to plants from other regions (*p <* 0.001) (Figure 3C). In our previous publication we associated yellow pixel percentage with the extend of tolerance to stress [6]. This indicated a difference between the coastal and central subpopulation in terms of responses to drought stress with the central being more tolerant. The control plants were maintained for a further eight weeks after which accessions were assessed for flowering. Only 24% of accessions from the coastal subpopulation showed evidence of flowering (all from region 2) compared to 53% of the central subpopulation (Figure 3D). This aligned with the slower flowering EDF^+^ phenotype which could be predicted to dominate the coastal subpopulation [10]. Too many of the plants that experienced drought stress subsequently died to allow the impact of stress on flowering to be determined.

### 2.4. Relating Climatic Niches to the Two Subpopulations in the Turkish Population of Brachypodium

**Figure 4:**
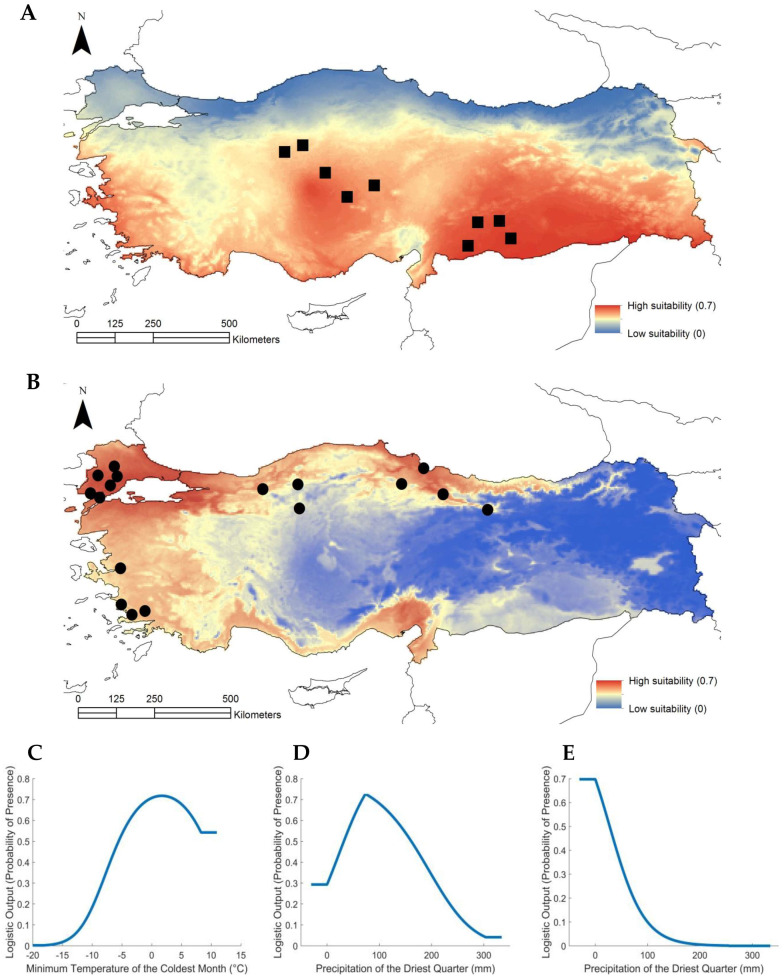
Bioclim modelling of environmental variables. These indicated the climate suitability of Brachypodium subpopulations in Turkey. (**A**) Color-coded, Maxent model climate suitability for the central subpopulation. Black squares represent sampling sites for the central subpopulation; (**B**) Maxent model climate suitability for the coastal subpopulation. Black circles represent sampling sites for the coastal subpopulation; (**C**) Maxent model response curve for the relationship between the probability of presence of the coastal subpopulation and the Minimum Temperature of the Coldest Month (°C); (**D**) Maxent model response curve for the relationship between the probability of presence of the coastal subpopulation and the Precipitation of the Driest Quarter (mm); (**E**) Maxent model response curve for the relationship between the probability of presence of the central subpopulation and the Precipitation of the Driest Quarter (mm).

Phenotypic, genetic and methylation analyses indicated that vernalization and drought tolerance could differentiate between the coastal and central subpopulation. Given this, we tested a series of environmental variables (“Bioclim”) to see if they aligned with the distribution patterns of the subpopulations. This analysis was based on the derivation of Maxent [28] climate niche models. Model fitting using the two Bioclim variables identified a beta multiplier of “2” as producing the most parsimonious models and this was used as the setting for the comparison against the null models. Comparing the Maxent against the null models showed that the central region subpopulation had a median area under the curve (AUC) >98 and the coastal subpopulation >97. The Maxent model for the central region subpopulation was influenced solely by the response to the “Precipitation of the Driest Quarter” and the response curve (Figure 4A) clearly showed that the probability of presence is at its highest in areas of low precipitation in the study area. The variable Minimum Temperature of the Coldest Month does not have any influence on the model. Instead, this variable was specific to coastal areas around the Black and Mediterranean Seas (Figure 4B). The Maxent model response curves show that the highest probabilities were observed towards the higher end of Minimum Temperature during the Coldest month (Figure 4C) and at mid-ranges of precipitation during the driest quarter (Figure 4D,E). The metrics for niche similarity computed for the two subpopulations (Supplementary Materials Figure S6) indicate that there is no significant climate niche overlap, compared to the pseudoreplicates from the pooled location points from the two subpopulations. This conclusion is supported by “Schoener’s D” (*p* = 0.01) and ‘I’ (*p* = 0.01). 

## 3. Discussion

In this study we used Brachypodium to examine how genetic and methylation variation could reflect climatic variation across Turkey. The natural selection of particular alleles is the foundation of evolutionary thinking, but the potential selective role of epigenetic features also needs to be considered [29,30]. There is some evidence that in mangrove plants (*Laguncularia racemosa*) epigenetic patterns can emerge in the absence of major genetic variation and these could be maintained for at least 20 years [14]. Other plant species growing in the presence of stress conditions can exhibit epigenetic variation which is not reflected at the genetic level [31,32]. There are various mechanisms through which epigenetic changes can influence phenotype, for example through CpG methylation of the promoter regions to influence gene expression [19,33] or when differential methylation present around TEs alters gene expression [34]. It is also difficult to untangle the possible contributions of genetic from epigenetic variation to a given phenotype. While genome level genetic and methylation changes can be closely associated as seen in collections of wild Swedish accessions of Arabidopsis and this appears to be equally the case with Brachypodium [9,21].

A recent study described the derivation of a new inbred population from Turkey to assess SNP and methylome variation [23]. This work indicated the close association between genetic and methylation patterns across the population. Phenotypic variation was mostly linked to genetic variation, but some CpG methylation appeared to be associated with some additional effects in certain environments. In this current paper, we also examined the genetic and methylome variation but across a wild-collected population where we used climatic information to govern our sampling strategy and experimental approach. This led to a clear clustering in our sampling sites (Supplementary Materials Figure S1) which differed from the more equidistant sampling sites used by researchers who characterized the other Turkish populations [22,23]. Other studies indicate an East-West split in Brachypodium genetic diversity across the Mediterranean, which is likely to reflect separate refugia from the last ice age [10,35]. Further, within the eastern population in Turkey, two subpopulations have already been described; variously designated EDF^+^ and T^+^ [10] or Subspecies A East and B East [9]. Crucially, these differences were not linked to geography. Our assessment reveals additional potential drivers of diversity within climate-environmental regions of Turkey; leading to our definition of central vs coastal subpopulations. Thus, one subpopulation was predominant in regions 1a and 1c and as a result was designated as central (belonging to the T^+^ cluster discussed above). In the other regions, 2, 3 and 4 the coastal (belonging to the EDF^+^ cluster) subpopulation was predominant. 

To preserve the methylome, seeds gathered from Turkish regions were assessed without undergoing generations of plant growth and meiosis in captivity. We did not use the inbred Turkish lines that are available e.g., [26] as epigenetic landmarks may have altered when propagated over many years [36]. We also used seeds directly sampled from Turkey but germinated in the UK under controlled environmental conditions. The germinated seedlings were used in our experiments in order to maintain, as much as possible, each accessions’ genome methylation status. Our methylomic assessments showed a similar separation of the Turkish accessions into coastal and central subpopulations. This was particularly prominent for CpG and CHG contexts but was also observable with CHH. In plants, CpG methylation is maintained by the methyltransferase MET1. However, CHG and CHH are methylated by CHROMOMETHYLASE2 and 3 (CMT2, CMT3) and DNA (cytosine-5)-methyltransferase (DRM2) [37]. DRM2 interacts with its target through an RNA-directed DNA methylation (RdDM) pathway which employs 24-nucleotide small interfering RNAs (24nt-siRNAs). This mechanism requires de novo modification that needs constant targeting by RdDM [38]. Both CMT2 and CMT3 are guided to their targets by histone H3 lysine 9 (H3K9) methylation [39,40]. CpG methylation is predominant in heterochromatic regions with TEs, other repeats as well as coding regions but CHG and CHH methylation is almost only found in heterochromatin [41,42]. Therefore, these different means of establishing the methylome are employed to confer the methylomes patterns that we observed. Eichten et al. [21] also found that the patterns of methylation in accessions were broadly similar but in our case that appeared to be a geographical split. Therefore, the relative role of genetic vs methylation in driving phenotypic differences is unclear. Our results might reflect a role of epigenetic changes in adaptation (for review, see [43]) to the two-contrasting climate-environmental regions mentioned above. This separation might be reinforced by the fact that both subpopulations are likely to harbor distinct sets of transposable element polymorphisms [44], which are strongly methylated, like in many other plant species. Recent assessments of transposable elements in a wild collection of Brachypodium suggest that there is no great variation in copy number so this is unlikely to explain the differences in global methylation seen in our populations [45].

The major differentiation between the two subpopulations could reflect the impact of allopatric separation by a barrier such as a mountain range. Thus, the coastal vs central subpopulation could have arisen from the highland Anatolian plateau which roughly corresponds to the location of the central subpopulation, reducing gene flow. This could be reinforced through localized adaptation to such as stress driving increased tolerance. Equally, reduced gene flow can also arise from a shift in flowering times which would influence the frequency of cross-pollination [9]. To investigate possible phenotypic differences between the coastal and central subpopulations, we employed phenomic approaches to assess drought responses in a diverse collection of Spanish accessions [6]. Eichten et al. [23] used manual approaches to measure plant height, third leaf length and width, tiller count, ear count, and flowering time. Our image analysis measurement concentrated on plant height, width and flowering time but additionally considered yellow pixels percentages as this was an indicator of chlorophyll loss, this being a symptom of stress. The major split seen between coastal and central subpopulations was not reflected in any measured growth characteristic but there was a difference in flowering time after vernalization and relative drought tolerance. The former aligned with the EDF^+^ phenotype which appeared to predominate in our coastal subpopulation although there was a significant proportion showing the rapid flowering type. 

Flowering time in Brachypodium is strongly associated with vernalization period [9] and drought self-evidently with precipitation, so we tested how far the two subpopulation sampling sites could be associated with relevant climatic features. This involved testing Bioclim models which best explained the distribution of the coastal and central subpopulations. The statistically significant association of the coastal subpopulation with the “lowest temperature of the coldest month” variable agreed with the coastal accessions having the EDF^+^ phenotype. Conversely, the distribution pattern of the central subpopulation was best explained by the “precipitation during driest quarter” Bioclim which would explain its greater degree of drought tolerance. These twin features could be major drivers of the marked genomic differences between the subpopulations. This stated the influence of flowering time as a barrier to gene flow in Brachypodium could be limited given its almost cleistogamous behavior which also results in a high degree of gene homozygosity [10]. Thus, the drought tolerance could play a much larger role in driving adaptive changes in the genome.

More detailed assessments are in progress; however, this current study highlights the importance of sampling strategies based on prevailing environmental conditions in order to better reveal differences between wild populations.

## 4. Materials and Methods 

### 4.1. Derivation of Turkish Lines

Single seed inflorescences were sampled from 55 Brachypodium accessions from five distinct Turkish environments. The sites are described in Table 1. This (T_0_) collection was transferred to Aberystwyth University, UK and three seeds from each accession were germinated under controlled environmental conditions (Levingtons F2 with horticultural grit [1/5 vol] added prior to use, 16 h photoperiod, natural light supplemented with artificial light from 400-W sodium lamps at 22 °C). After six weeks the first three leaves were collected from each accession and frozen in liquid N_2_ prior to DNA extraction. 

### 4.2. Whole Genome Sequencing and Single Nucleotide Polymorphism Calling

Genomic DNA was isolated from 10–15 mg of leaf tissue using the cetyl trimethylammonium bromide (CTAB) method [46]. Sequencing libraries were constructed with Illumina TruSeq Nano DNA kit and sequenced using Illumina X-Ten at 10× genome coverage (Macrogen Inc., Seoul, Republic of Korea; quality control (QC) data are provided in Supplementary Materials Table S4) For each accession sequenced paired-end reads were aligned using the BWA-MEM algorithm of Burrows-Wheeler Aligner (v.0.7) [47] to version 3.1 of the Brachypodium reference genome on Phytozome (https://phytozome.jgi.doe.gov/). After removing duplications with Sambamba (v.0.6.8) [48], SNPs were called with the Genome Analysis Toolkit (GATK, v.4.0.2.1) and filtered for quality scores lower than 20, a mean depth lower than 50 and a StrandOddsRatio higher than three. From the SNP set obtained synonymous SNPs were extracted through annotating to the reference *Brachypodium distachyon* synonymous positions in the genome (v.3.1), LD-pruned and filtered for minor allele frequency of 0.05. This generated a final data set of 5021 SNPs. To assess the genetic structure in 55 accessions, a phylogenetic tree was derived using HDClusters in the SNPrelate package (v.1.22.0) [49]. Population genetic and TESS3 structure analyses were performed with the tess3r package (v.0.1) implemented in R [50]. To obtain a wider depiction of genetic clustering, together with our individuals, using GATK (v.4.0.2.1) we combined whole genome sequences (vcf files) of accessions that were previously analyzed by Gordon et al. [10]. The merged file was later annotated to the synonymous position in the reference genome (annotation v.3.1), filtered and LD-pruned as described above. In total we obtained 5792 LD-pruned synonymous SNPs with no missing data. The cross-validation plot for the structure analysis was done using tess3r (v.01). All genomic data are available from https://www.ncbi.nlm.nih.gov/sra/PRJNA605320.

### 4.3. Bisulfite Sequencing and Data Analysis

Genomic DNA was isolated and BS-Seq libraries were constructed with Illumina TruSeq DNA methylation kit. After sequencing, poor quality reads and adapters were removed using TrimGalore! (http://www.bioinformatics.babraham.ac.uk/projects/trim_galore/, accessed 10 September 2020). Trimmed reads were mapped to the *B. distachyon* v.3.1 chloroplast and genomic reference sequences (QC, Supplementary Materials Table S5). After removing of duplicated reads with Bismark (v.1.3) [51], cytosine methylation in CpG, CHG and CHH contexts was estimated as an average over two duplicates for each accession (Supplementary Material Table S2). Epigenetic structure together with methylation and coverage statistics for each sample and context were performed using methylKit (v.1.8.1) [52] and GenomicRanges (v.1.34) within Bioconductor [53]. Conversion rates for whole-genome BS-Seq data in all three contexts are shown in Supplementary Materials Table S2.

### 4.4. Phenomic Experiments 

To provide sufficient plants for the phenotyping experiments, seeds of second generation (T_1_) were used. Seeds of the collected accessions were germinated in pots with 50 g of 4:1 Levington F2: grit sand. After two weeks, seedlings were vernalized for a further six weeks, and then the eight-week-old plants were transferred into the plant screening system (National Plant Phenomics Centre, NPPC, Aberystwyth, UK). The NPPC allows computer regulated watering of each individual plant and watering was withdrawn from four replicates from each genotype to achieve 15% soil water content (SWC) by seven days. This level of SWC was maintained for 12 days; the end of the experiment, the remaining replicates continued to be watered to 75% SWC. Images were captured at 12 days after watering was first restricted using a single-lens reflex camera Nikon D60 (Nikon Corporation, Tokyo, Japan) with an 18–55 mm lens. For uniform processing results and further analysis, images were processed to generate 24-bit RGB color images where each channel had 256 class color levels. Images were segmented from background in RGB color space. Plant growth parameters and color pixel data were extracted as plant height, top view and side view projection area and color information were extracted from the processed images using C++ (Visual Studio 2012) and Open Source Computer Vision Library (Open CV, v.2.4.9) [6]. Yellow pixel percentages as a proportion of total pixel numbers were calculated. Derived phenotype data were subjected to ANOVA using SPSS (v.25) software and residual plots were inspected to confirm normality of the distribution. Significance of differences between means was determined by contrast analysis (Scheffe’s).

### 4.5. Climate Modelling 

Potential differences between the climate niches of the different subpopulations by the multi-omic analyses were investigated by using the Identity test as described by Warren et al. [54]. This entailed creating Maxent [28] climate niche models for both subpopulations. We undertook two approaches for selecting predictor climate variables for the models. A PCA approach was undertaken to condense the variance from 19 Bioclim climate variables from the WorldClim climate dataset [55] into a smaller number of principal components. Having identified the final fitted models, we then tested the ability of the models to identify significant associations between the distribution of the subpopulations and the climate variables. This was undertaken by using a method proposed by Beale et al. [56,57] and developed by Williams et al. [58] where null models retaining the spatial structure of the presence points are used to compare against the real models.

Climate niche models that identified significant associations between the distribution of the subpopulations and the climate variables were run with ENMtools (v.1.3) [55] with the beta multiplier settings identified during the model fitting stage. One hundred pseudoreplicates were created for the Identity test from presence points of both subpopulations which yielded 100 values for ‘I’ and Schoener’s D [54] to be compared against the values from the observed models. The hypothesis of niche identity was rejected if more than 95 of the pseudoreplicates had niche overlap values in excess of the niche overlap values from the observed subpopulations. 

## 5. Conclusions

Defining variation amongst populations is important to determining likely evolutionary pressures shaping natural selection. In this study, we use the well-established model grass—Brachypodium—to define variation in Turkish populations. Crucially, our sampling strategy was biased towards representing the major climatic regions of Turkey. Variation was characterized at the genetic and methylome levels, to reflect two of the levels at which selection could act. Both genetic and methylome variation suggested two subpopulations which we designated as coastal and central. Phenotypic assessment suggested the subpopulations exhibited, respectively, a preponderance of differential flowering and drought tolerance phenotypes. This aligned with Bioclim models which suggested that late flowering was linked to the cold month—i.e., vernalization—and drought with relative precipitation in the driest month. Therefore, we provide evidence of climate being associated with genetic and methylome variation. Although Turkey has been extensively sampled by others, our environment bias sampling approach has provided important insights into potential drivers of Brachypodium evolution.

## Figures and Tables

**Table 1:** Geographical origins of Brachypodium accessions used in this study (sorted by collecting date).

Region	Station	Collecting Date	Latitude	Longitude	Altitude (m) *
2	1	22-May-2016	38.5055	27.31671667	349
2	5	23-May-2016	37.49243333	27.3395	67
2	8	24-May-2016	37.20506667	27.65306667	40
2	9	24-May-2016	37.31233333	28.03705	626
2	14	25-May-2016	36.94201667	30.96305	10
2	20	27-May-2016	36.95461667	34.7507	161
1c	25	28-June-2016	39.86911667	32.7329	1042
1c	26	28-June-2016	39.68008333	32.19811667	879
1c	27	29-June-2016	38.40685	34.03873333	1122
1c	28	30-June-2016	38.738	34.83881667	1063
1a	29	1-July-2016	37.73385	38.53376667	668
1a	30	1-July-2016	37.69656667	37.89476667	696
1a	31	2-July-2016	37.03268333	37.60995	735
1a	32	2-July-2016	37.23601667	38.87008333	605
1c	34	3-July-2016	39.09433333	33.39311667	933
1c	35	3-July-2016	40.19286667	32.59326667	1059
4	36	3-July-2016	40.73106667	31.51755	865
3	38	7-July-2016	41.12035	26.65313333	58
3	40	23-July-2016	40.61361667	26.43273333	63
3	41	27-July-2016	41.0926	27.22096667	97
3	42	8-August-2016	41.3691	27.13661667	50
4	45	15-August-2016	40.86231667	32.54991667	1242
4	47	16-July-2016	40.87441667	35.60698333	605
4	49	16-July-2016	40.59275	36.83505	283
4	51	16-July-2016	40.15375	38.14713333	920
4	52	19-July-2016	41.32165	36.25826667	128
3	54	29-July-2016	40.83846667	27.02001667	205
3	55	29-July-2016	40.5003	26.70376667	88

Five distinct regions (1a, 1c, 2, 3, and 4) were selected for Brachypodium sampling defined by Köppen climate classifications [25]. There were at least five sampling sites (“stations”) within each region and individual stations were sampled at least 12 times to derive individual accessions. Thus, for example, an accession designed 2_14_13 refers respectively to region, station, individual plant sample. *—meters above sea level.

## Supplementary Materials

Supplementary materials can be found at https://www.mdpi.com/1422-0067/21/18/6700/s1. Figure S1. Climatic regions of Turkey and Brachypodium sampling sites. Figure S2. Principal component analyses of SNP variation in the Brachypodium accessions. Figure S3. Ancestry coefficient assessment of structure analysis. Figure S4. Exemplar RGB image analysis of Brachypodium accessions in the National Plant Phenomics Centre, Aberystwyth, UK. Figure S5. Phenotypic variation in the Turkish collection of Brachypodium. Figure S6. Climate niche overlap metrics for Brachypodium subpopulations in Turkey. Table S1. Classification of Brachypodium accessions used in this study. Table S2. Conversion rates for whole genome bisulfite sequencing data in CpG, CHG and CHH context. Table S3. Phenotypic data generated at the National Plant Phenomic Centre (UK). Table S4. Whole genome sequencing quality check results. Table S5. Bisulfite sequencing quality check results.
