# Supplementary material for: Genetic and Methylome Variation in Turkish Brachypodium Distachyon Accessions Differentiate Two Geographically Distinct Subpopulations
Source: Int J Mol Sci. 2020 Sep 13;21(18):6700. doi: 10.3390/ijms21186700 (PMC7556024; doi:10.3390/ijms21186700)
Supplement: Supplementary file 1 [file ijms-21-06700-s001.zip › ijms-21-06700-supplementary figures.pdf]

International Journal of
Molecular Sciences

MDPI

Supplementary Materials

Article Title: Genetic and Methylome Variation in Turkish *Brachypodium Distachyon* Accessions Differentiate Two Geographically Distinct Subpopulations

Authors: Aleksandra Skalska, Christoph Stritt, Michele Wyler, Hefin W. Williams, Martin Vickers, Jiwan Han, Metin Tuna, Gulsemin Savas Tuna, Karolina Susek, Martin Swain, Rafał K. Wóycicki, Saurabh Chaudhary, Fiona Corke, John H. Doonan, Anne C. Roulin, Robert Hasterok and Luis A. J. Mur

The following Supplementary Materials are available for this article:

Supplementary Figures

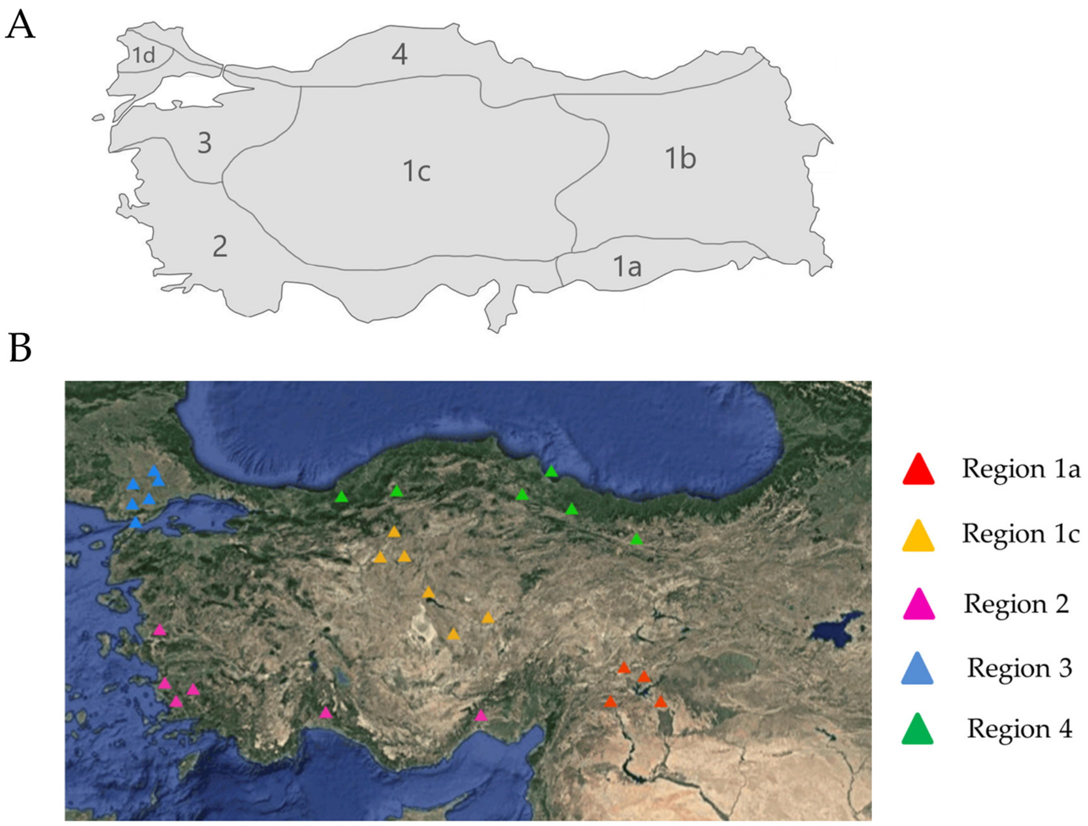

Figure S1. Climatic regions of Turkey and Brachypodium sampling sites. (**A**) Köppen classifications of Turkey into climatic regions. Region 1 is sub-divided into 1a, 1b and 1c to reflect the impact of the Armenian highlands mountain range; (**B**) Brachypodium sampling sites chosen to reflect the climate classifications shown in (**A**).

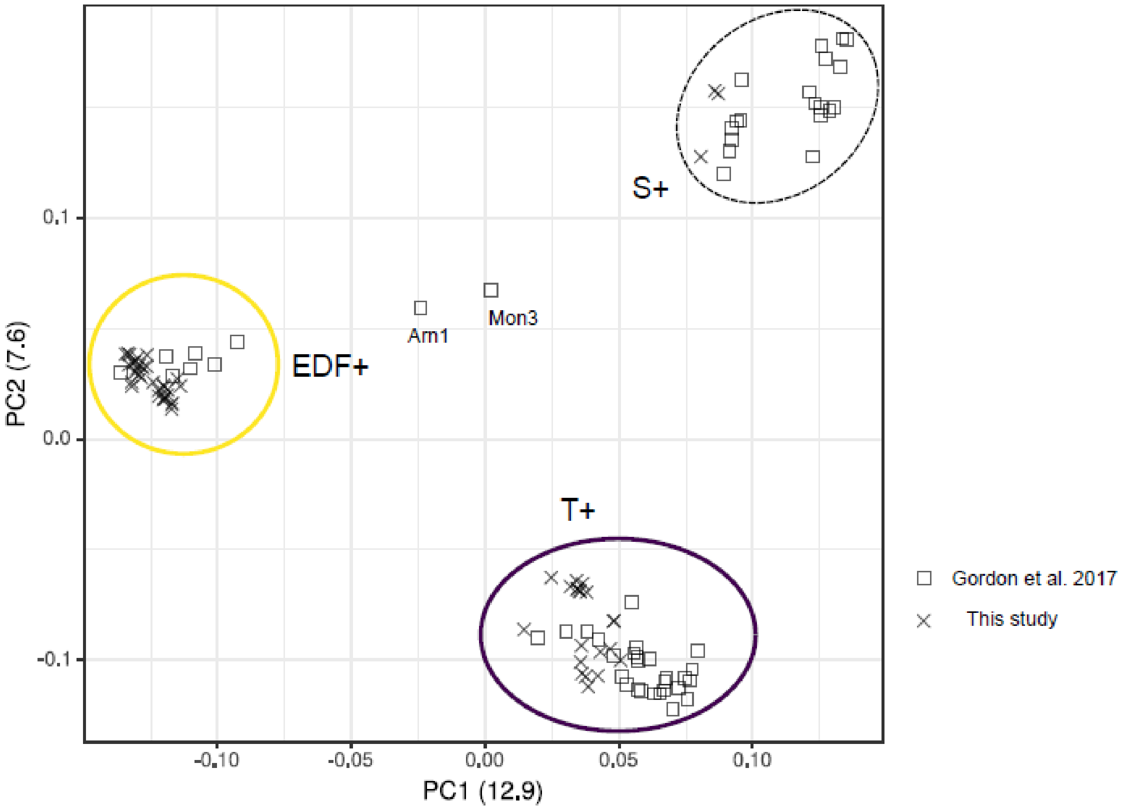

Figure S2. Principal component analyses (PCA) of 5,792 unlinked SNPs at synonymous positions linked to 111 Brachypodium accessions consisting of accessions collected as part of this current study and the "T+" (Turkish), "S+" (Spanish) and the EDF+ (Extremely Delayed Flowering Phenotypes) previously defined by Gordon et al. 2017 [10]. The ellipses define 95% confidence intervals for each group and relate to our definition of coastal (yellow) and central (purple) subpopulations. Arn1 and Mon3 represent two Spanish admixed accessions.

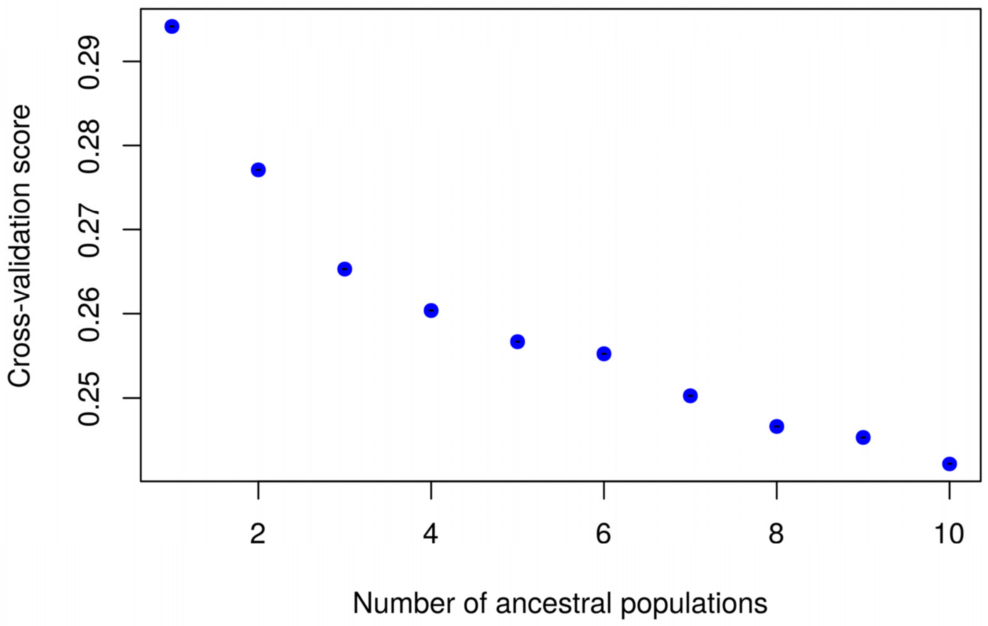

Figure S3. Ancestry coefficient assessment of TESS3 analysis indicated that variation was best explained by two (K) groups.

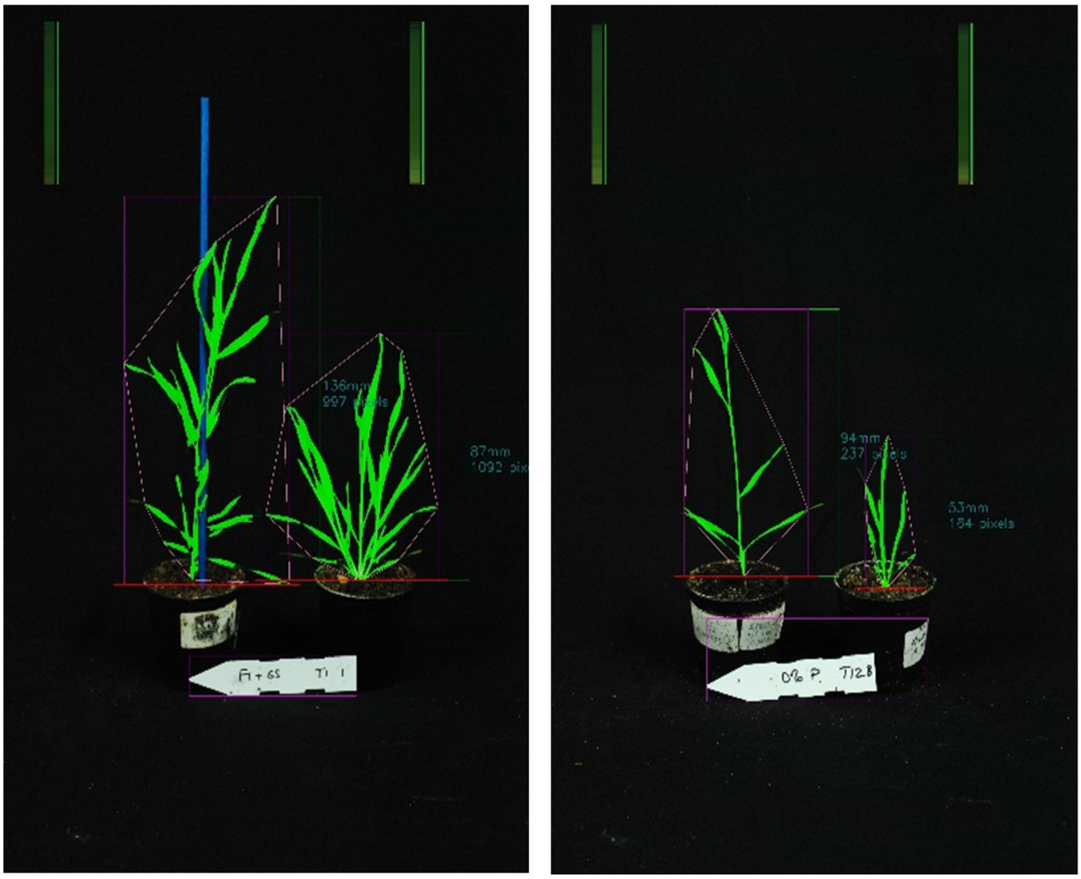

Figure S4. Exemplar RGB image analysis of Brachypodium accessions in the National Plant Phenomics Centre.

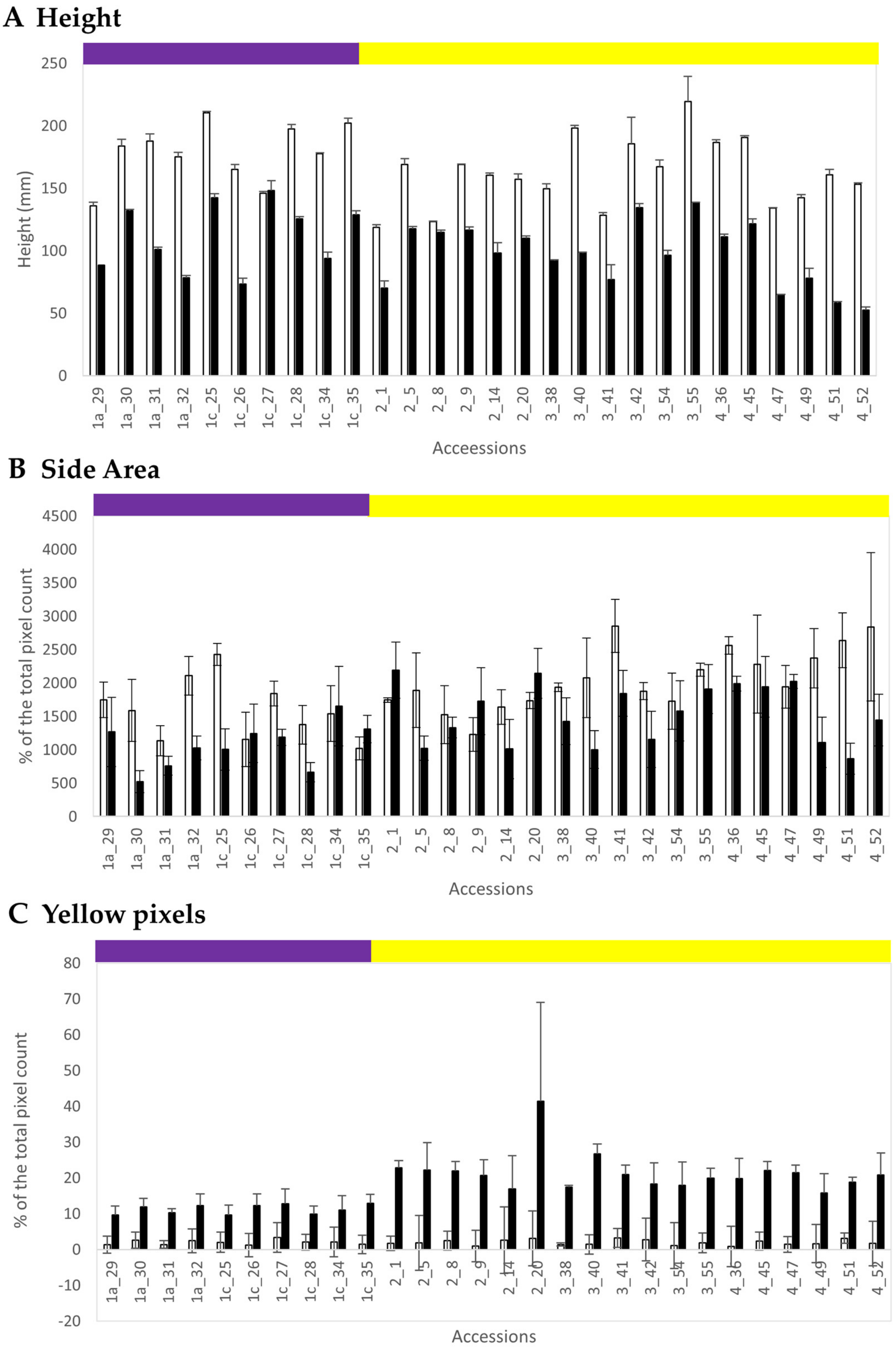

Figure S5. Phenotypic variation in the Turkish collection of Brachypodium. Brachypodium accessions (n = 8 plants) were vernalised for six weeks at 4 °C before being transferred to 22 °C and either maintained with full watering (n = 4 plants; white bars) or at 15% soil water content (n = 4 plants, black bars). At 12 d the plants were imaged at the National Plant Phenomics Centre, Aberystwyth, UK, where (**A**) height and **(B)** side area were derived; (**C**) yellow pixels were extracted from the images of plants. Pixel data are presented as % of the total pixel count for the whole plant. The purple and yellow horizontal bars on (**A**), (**B**) and (**C**) indicate data from accessions sampled from central (1a, 1c) and coastal (2, 3, 4) subpopulations, respectively.

A

B

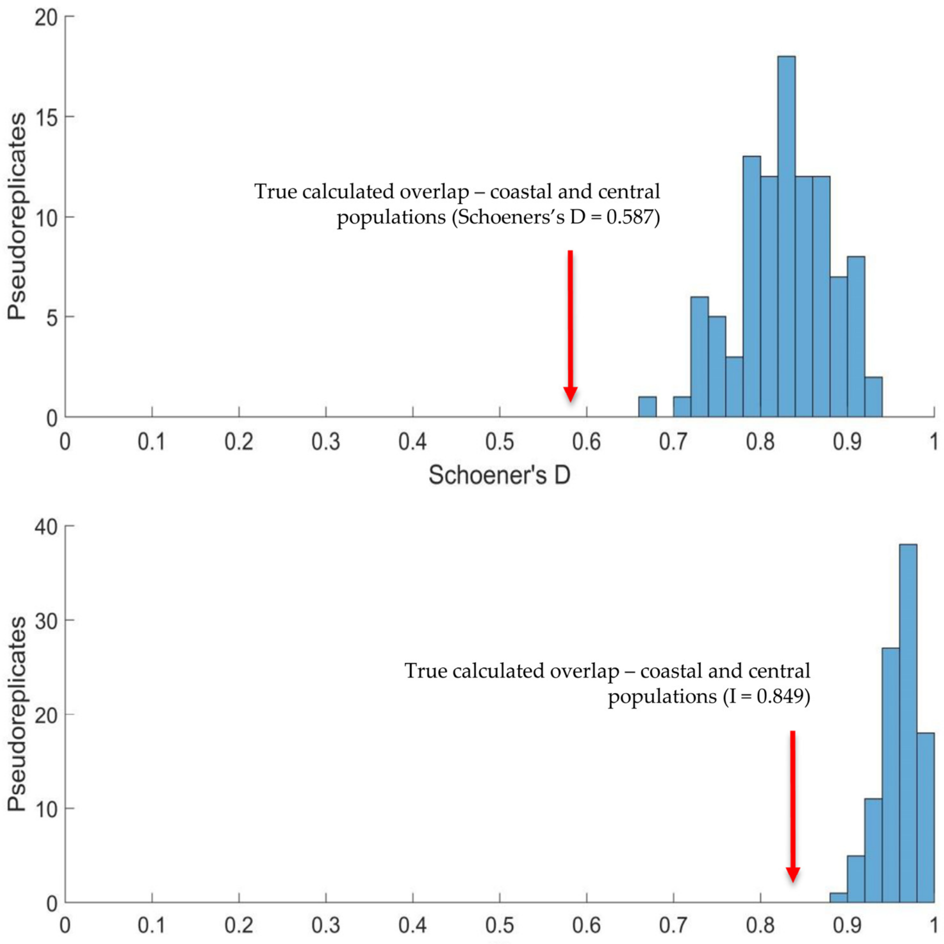

Figure S6. Climate niche overlap metrics for Brachypodium subpopulations in Turkey. Arrows represent the calculated niche overlap values for the two Brachypodium subpopulations as identified by our multi-omic analysis for two climate niche overlap metrics (**A**) "Schoener's D" and (**B**) "I". Columns represent the niche overlap values from 100 pseudoreplications from the ENM tools Identity test where all samples were pooled, and their population identities randomised. The niche overlap values calculated from our identified subpopulations were far outside the range of values from the pseudo-replications from pooled samples for both niche overlap metrics (**A**) "Schoener's D" and (**B**) "I". Thus, the climate niches of the two subpopulations are more different than expected by chance (p = 0.01).
